# Probability of Extinction and Peak Time for Multi-Type Epidemics with Application to COVID-19 Variants of Concern

**DOI:** 10.1016/j.jtbi.2025.112135

**Published:** 2025-04-28

**Authors:** Jacob Curran-Sebastian, Louise Dyson, Edward M Hill, Ian Hall, Lorenzo Pellis, Thomas House

**Affiliations:** 1 Section of Epidemiology, Department of Public Health, University of Copenhagen, Copenhagen, Denmark; 2 School of Life Sciences and Mathematics Institute, University of Warwick, Coventry, UK; 3 The Zeeman Institute for Systems Biology & Infectious Disease Epidemiology Research, University of Warwick, Coventry, UK; 4 Civic Health Innovation Labs and Institute of Population Health, University of Liverpool, Liverpool, UK; 5 NIHR Health Protection Research Unit in Gastrointestinal Infections, University of Liverpool, Liverpool, UK; 6 Department of Mathematics, University of Manchester, Manchester, UK

## Abstract

During the COVID-19 pandemic, the emergence of novel variants of concern (VoCs) prompted different responses from governments across the world aimed at mitigating the impacts of more transmissible or more harmful strains. We model the invasion of a novel VoC into a population with heterogeneous vaccine- and infection-acquired immunity using a multi-type branching process framework with immigration. We define the number of cases needed to be reached to ensure stochastic extinction of this strain is unlikely and, therefore, the strain has become established in the population. To estimate the first-passage time distribution to reach this number of cases we use a mixture of stochastic simulations and analytic results. The first-passage time distribution gives a time window that is useful for policymakers planning interventions aimed at suppressing or delaying the introduction of novel VoC. We apply our method to a model of COVID-19 in the United Kingdom, though our results are applicable to other pathogens and settings.

## 1 Introduction

The emergence of SARS-CoV-2 in December 2019, followed later by novel variants of concern (VoCs), created a focus on border controls and nationwide lockdowns as a defence against importing infected cases into a population. Border restrictions are known to have only limited effectiveness in delaying the ingress of infected cases [18, 27], but the ability of an imported strain to establish itself in a society is complicated by the levels of non-pharmaceutical interventions (NPIs) in place and by the extent of the vaccination programme coverage. Taking into account both the external infection pressure and the internal disease dynamics results in a large degree of uncertainty when estimating the time taken for a novel strain to become established. However, estimating the duration between the ingress of a novel strain and its establishment in the population, as well as the uncertainty in this duration, is crucial for policymakers to be able to plan appropriate interventions, and to evaluate the relative benefits of different policies.

Branching processes are widely applied in epidemic modelling, and have been used during the COVID-19 pandemic to model the early behaviour of outbreaks in homogeneously mixed populations [23, 4] as well as to understand the extent and nature of super-spreading events [11]. These processes are often analytically tractable, and offer insights into the probability that an outbreak will grow to a large size, which can in turn be used for planning mitigation strategies (see Southall et al. [30] for a recent review of methods for estimating the risk of a large outbreak). More structured branching process models have also been used to understand household transmission and the effectiveness of contact tracing [14].

We use a multi-type branching process model to understand the stochastic invasion of a VoC into a population with an ongoing vaccination programme and where part of the population exhibits immunity due to prior infection with a resident strain. We use this framework to obtain the probability that an imported strain ultimately becomes established in the population, as well as the mean and variance in the number of cases over time. Furthermore, we quantify the time after which the system’s dynamics can be well-approximated by a deterministic model, at which point the epidemic grows exponentially. We interpret this as the time taken for the novel strain to become fully established in the population, after which point further restrictions need to be put in place at a population level in order for the epidemic to be controlled. We approximate the uncertainty around this time scale by using a one-dimensional diffusion approximation to the multi-type branching process, which has an analytic solution, and we compare these results with those obtained via stochastic simulations. Finally, we propagate this uncertainty forwards in time using a hybrid stochastic-deterministic model to quantify the corresponding uncertainty in the timing of the peak of the epidemic.

## 2 Methods

### 2.1 Multi-type Branching Process Model with Heterogeneous Immunity

We model the disease dynamics of a novel VoC entering into a population that has had previous exposure to a resident variant and has undergone an incomplete vaccination programme. We assume that existing immunity in the population due to either prior infection or vaccination might not be totally effective at blocking infection by the novel VoC. We model the early phase of this outbreak using a continuous-time multi-type Galton-Watson branching process; many of the results relevant to our analysis are given in [9].

We consider an epidemic for which infected cases are divided into 2*m* types, consisting of *m* exposed types and *m* infectious types (so that, in the language of branching processes, we have *m types-at-birth* [7, Ch. 6]). The distinction between different *types-at-birth* corresponds to heterogeneous profiles in the susceptibility and infectiousness of different groups within the resident population. For the particular application we are interested in, we only need to consider heterogeneous susceptibility resulting from prior infection with a heterologous strain and from a partial vaccination programme, and so will assume that infectious individuals of all type have equal infectivity. In essence, this means that we will consider 4 susceptibility profiles, corresponding to individuals in the population who are completely susceptible, previously infected but not vaccinated, vaccinated but not previously infected, or neither vaccinated nor previously infected. However, we will keep the notation general in what follows.

We denote the rate at which an infectious individual of type *j* infects any susceptible individual of type *k* by *β*_*jk*_ and the recovery rate (assumed to be the same for infectious individuals of all *types-at-birth*) by *γ*. We further denote the rate at which an individual who has been exposed to infection becomes infectious by *δ*, which is again assumed to be common to all *types-at-birth*.

Following [9], we denote the rate at which the next event occurs (either end of incubation period, infection event or recovery) for a case of type *j* by *ω*_*j*_ for 1 ≤ *j* ≤ *m*. The rate at which an individual of type 1 ≤ *j* ≤ *m* leaves their incubation period is given by *ω*_*j*_ = *δ*, and the rate at which an infectious individual of type *m* + 1 ≤ *j* ≤ 2*m* either infects a susceptible individual or recovers is given by $\omega_{j}=\sum_{k=0}^{m}\beta_{jk}+\gamma$. Note that, for the application considered in this work, where all individuals are equally infectious, we have that *β*_*jk*_ = *β*_*k*_ for all *j*, so that *ω*_*j*_ has the same value for all *m* + 1 ≤ *j* ≤ 2*m*. Note, further, that here *β*_*k*_ includes the proportion of susceptible individuals that belong to *type-at-birth k*, i.e. if we denote this proportion as *S*_*k*_ (in line with the application presented in Section 2.5, where we work in terms of fractions of the total population, rather than in number of individuals), we have *β*_*jk*_ = *β*_*k*_ = *βS*_*k*_, where *β* is the baseline transmission rate common to all infectious individuals of *type-at-birth j*.

Let $\text{s}={\left(s_{j}\right)}_{j=1}^{2m}$ be a vector of length 2*m*, and let ***ξ***_*j*_ be the random vector (also of length 2*m*) of individuals produced by an individual of type *j*. The probability generating function (PGF) for the offspring distribution of an individual of type *j*, denoted $P_{j}\left(\text{s}\right),$ is defined as:

(1) $$P_{j}(\text{s})=\overset{\infty }{\underset{n_{1},n_{2},\ldots ,n_{2m}=0}{\sum }}P\left[{\text{ξ}}_{j}=\text{n}\right]s_{1}^{n_{1}}\times \cdots \times s_{2m}^{n_{2m}},$$

where ***n*** = (*n*_1_, … *n*_2*m*_) is a vector, and *n*_*k*_ refers to the number of offspring of type *k*. In the above expression, we sum over all possible combinations of types of offspring that an individual of type *j* can have.

For 1 ≤ *j* ≤ *m*, an individual of type *j* is exposed but not yet infectious. When they end their incubation period, they become an infectious individual of the same *type-at-birth*, and thus produces a single individual of type *j* + *m* (and no others of any type) with probability 1. For *m* + 1 ≤ *j* ≤ 2*m*, an individual of type *j* is infectious, and infects another individual of *type-at-birth k* for 1 ≤ *k* ≤ *m* with probability *β*_*jk*_*/ω*_*j*_, producing both an infectious individual of type *j* (an identical copy of itself) and a new exposed individual of type *k*. Otherwise, the infectious individual recovers with probability *γ/ω*_*j*_ and produces no individuals of any type. In this sense, each new generation of the branching process contains individuals produced via some event experienced by the previous generation, namely via the end of an incubation period, the transmission from an infectious individual, or the replacement of an infectious individual by an identical copy of itself that occurs when that individual infects a susceptible person.

For each type *j*, the PGF for the offspring distribution is therefore given by:

(2) $$P_{j}(\text{s})=\{\begin{array}{l} s_{j+m}\;\text{for}\;1\leq j\leq m,\\ \frac{\sum_{k=0}^{m}\beta_{k}s_{k}s_{j}}{\omega_{j}}+\frac{\gamma }{\omega_{j}}\;\text{for}\;m+1\leq j\leq 2m. \end{array}$$

We first consider a process that starts only with a single case of type *i* at time *t* = 0, in order to obtain the probability of extinction for a lineage arising from a single index case. For this process, we consider the vector ${\text{Z}}^{\left(i\right)}(t)=\left(Z_{j}^{\left(i\right)}(t)\right)(1\leq i,j\leq 2m)$ where $Z_{j}^{\left(i\right)}(t)$ denotes the number of cases of type *j* at time *t* for a process starting with a single case of type *i*. For each *i*, the random variable ${\text{Z}}^{\left(i\right)}(t)$ has PGF:

(3) $$Q_{i}(t,\text{s})=\overset{\infty }{\underset{n_{1},n_{2},\ldots ,n_{2m}=0}{\sum }}\mathrm{Pr}\left({\text{Z}}^{\left(i\right)}(t)=\text{n}\right){\text{s}}^{\text{n}}.$$

The notation ***s***^***n***^ denotes the product $s_{1}^{n_{1}}\times \cdots \times s_{2m}^{n_{2m}}$.

For each *i*, the generating functions $Q_{i}\left(t,\text{s}\right)$ satisfy the backward Kolmogorov equations:

(4) $$\frac{\partial Q_{i}(t,\text{s})}{\partial t}=-\omega_{i}\left[Q_{i}(t,\text{s})-P_{i}(\text{Q}(t,\text{s}))\right],\;\text{subject}\;\text{to}\;\text{Q}(0,\text{s})=\text{s},$$

where $\text{Q}(t,s)={\left[Q_{i}(t,s)\right]}_{i=1}^{2m}$ is a vector. Setting ***s*** = **0** gives the extinction probabilities $q_{i}\left(t\right)=Q_{i}\left(t,\text{0}\right),$ i.e the probabilities that, by time *t*, there are no cases of any type for a process starting with a single particle of type *i*.

In addition to the process described above, we also consider the process that begins with no cases of any type, but that allows importation of cases of type *i* for 1 ≤ *i* ≤ 2*m* at a constant rate *η*_*i*_. We denote the vector of the number of cases of each type at time *t* for this process by ***Y*** (*t*) = (*Y*_*j*_(*t*)) (1 ≤ *j* ≤ 2*m*). Once cases have been imported and the outbreak has begun, individuals behave according to exactly the same rules as in the importation-free process (where the number of cases was instead denoted by ${\text{Z}}^{\left(i\right)}(t)$), with each individual of type *j* producing a random vector of individuals ***ξ***_*j*_ as before. We define the sum *η* =∑_*i*_ *η*_*i*_, and the vector ***η*** = (*η*_*i*_). ***Y*** (*t*) has the associated generating function:

(5) $$R(t,\text{s})=\overset{\infty }{\underset{n_{1},n_{2},\ldots ,n_{2m}=0}{\sum }}\mathrm{Pr}(\text{Y}(t)=\text{n}){\text{s}}^{n}.$$

We obtain *R*(*t*, ***s***) by solving the following equation, given in [9]:

(6) $$\frac{\partial R(t,\text{s})}{\partial t}=-\eta R(t,\text{s})+\overset{2m}{\underset{i=0}{\sum }}\eta_{i}R(t,\text{s})Q_{i}(t,\text{s}),\;\text{subject}\;\text{to}\;R(0,\text{s})=\text{1}.$$

Again setting ***s*** = 0, we find the probability, *r*(*t*) = *R*(*t*, **0**), that there are no cases of any type at time *t*. In the case when immigration is present, if the process is supercritical (i.e. the maximum eigenvalue of the matrix **Ω** defined in Equation (8) below is greater than zero), we note that lim_*t*→∞_ *r*(*t*) = 0 and hence the probability of having zero cases becomes arbitrarily small. This is because, with immigration, new cases are allowed to enter the population, and hence the state ***Y*** (*t*) = **0** is no longer absorbing for any *t*. This means that we should no longer interpret *r*(*t*) as a probability of extinction, though we may interpret *r*(*t*) *<<* 1 loosely as evidence that ***Y*** (*t*) is sufficiently bounded away from zero at time *t*.

Denote by ${\text{μ}}_{{\text{Z}}^{\left(i\right)}}(t)=E\left[{\text{Z}}^{\left(i\right)}(t)\right]={\left(E\left[Z_{j}^{\left(i\right)}(t)\right]\right)}_{j=1}^{2m}$ the vector of the mean number of cases of each type at time *t* in the outbreak without importation (and a single initial case of type *i*) and, similarly, denote by ${\text{μ}}_{\text{Y}}(t)=E[\text{Y}(t)]={\left(E\left[Y_{j}(t)\right]\right)}_{j=1}^{2m}$ the mean vector for the outbreak with importation (and no initial case). These vectors satisfy the equations:

(7) $$\frac{\text{d}{\text{μ}}_{{\text{Z}}^{\left(i\right)}}}{\text{d}t}=\Omega {\text{μ}}_{{\text{Z}}^{\left(i\right)}}(t)\;\text{subject}\;\text{to}\;{\text{μ}}_{{\text{Z}}^{\left(i\right)}}(0)={\text{Z}}^{\left(i\right)}(0)$$

(8) $$\frac{\text{d}{\text{μ}}_{\text{Y}}}{\text{d}t}=\Omega {\text{μ}}_{\text{Y}}(t)+\text{η}(t)\;\text{subject}\;\text{to}\;{\text{μ}}_{\text{Y}}(0)=\text{Y}(0),$$

where the matrix **Ω** has (*i, j*)^th^ entry given by ${\text{Ω}}_{i,j}=\frac{\partial P_{i}}{\partial s_{j}}(1)-\omega_{i}\delta_{i,j}$, with 1 ≤ *i, j* ≤ 2*m*, and where *δ*_*i,j*_ is the Kronecker delta.

Let the scalar random variables $Z^{\left(i\right)}(t)=\sum_{j=1}^{2m}Z_{j}^{\left(i\right)}(t)$ and $Y(t)=\sum_{j=1}^{2m}Y_{j}(t)$ denote the total number (i.e. the sum) of individuals of any type in the processes without and with importation, respectively. Then the scalar-valued means for the number of cases of any type at time *t* in the processes without and with importation, respectively, are defined as $\mu_{Z^{\left(i\right)}}(t)=\sum_{j=1}^{2m}{\left({\text{μ}}_{{\text{Z}}^{\left(i\right)}}(t)\right)}_{j}$ and $\mu_{Y}(t)=\sum_{j=1}^{2m}{\left({\text{μ}}_{\text{Y}}(t)\right)}_{j}$.

We obtain expressions for the time-dependent variance of this process following the derivation in [9], which was also demonstrated in [24]. We consider a population that, at least initially, begins with no cases infected with the invading strain so that any outbreak is seeded by cases imported from outside the population. At time *t*, the variance-covariance matrix for the number of cases is given by Var(***Y*** ^(*i*)^(*t*)) = 𝔼[***Y*** ^(*i*)^(*t*)^*T*^ ***Y*** ^(*i*)^(*t*)] − 𝔼 [***Y*** ^(*i*)^(*t*)]^*T*^ 𝔼 [***Y*** ^(*i*)^(*t*)]. The superscript *i* here denotes the process for which only cases of type *i* are imported. For such a process, Var(***Y*** ^(*i*)^(*t*)) reduces to calculating the integral:

(9) $$\text{Vec}\left(\text{Var}\left({\text{Y}}^{\left(i\right)}(t)\right)\right)=\int_{0}^{t}\eta_{i}\left[\text{Vec}\left[{\text{V}}_{i}(t-\tau )\right]+{\text{e}}^{(t-\tau )\Omega T}⊗{\text{e}}^{(t-\tau )\Omega T}\text{Vec}\left[{\text{u}}_{i}{\text{u}}_{i}^{T}\right]\right]\text{d}\tau .$$

Here we have made use of the Vec operator, which stacks the columns of an *m* × *n* matrix to produce a vector of length *mn* and ⊗ denotes the Kronecker product. ***V***_*i*_(*t*) is the variance-covariance matrix at time *t* of the immigration-free process that begins with a single case of type *i*, i.e. it is the variance of the process ${\text{Z}}^{\left(i\right)}(t)$. The vector ***u***_*i*_ is the standard basis vector whose *i*^th^ component is equal to 1 and all other components are equal to zero.

Splitting (9) into the sum of two integrals, we first write:

(10) $$\int_{0}^{\tau }{\text{e}}^{(t-\tau )\Omega^{*}}⊗{\text{e}}^{(t-\tau )\Omega^{*}}\text{d}\tau =\varphi_{1}\left(\Omega^{*}⊕\Omega^{*}\right),$$

where ***A*** ⊕ ***B*** is the Kronecker sum of an *r* × *r* matrix ***A*** and an *n* × *n* matrix ***B*** given by ***A*** ⊕ ***B*** = ***A*** ⊗ ***I***_*n*_ + ***I***_*r*_ ⊗ ***B*** and *φ*_1_ is the matrix exponential integrator function given by:

(11) $$\varphi_{1}(\text{A})=\left(\exp(\text{A})-{\text{I}}_{r}\right){\text{A}}^{-1},$$

for an invertible *r* × *r* matrix ***A*** [17].

For the first part of the integral (9) we make use of the fact that

(12) $$\text{Vec}\left[{\text{V}}_{i}(t)\right]=\left(u_{j}⊗\text{I}⊗\text{I}\right)\int_{0}^{t}{\text{e}}^{s\Omega }⊗{\text{e}}^{(t-s)\Omega *}⊗{\text{e}}^{(t-s)\Omega *}\text{ds,}$$

so that, substituting *τ* for *t* − *τ* we have:

$$\int_{0}^{t}\text{Vec}\left[{\text{V}}_{i}(t-\tau )\right]\text{d}\tau =\left({\text{u}}_{i}⊗\text{I}⊗\text{I}\right)\left(\int_{0}^{t}\int_{0}^{\tau }{\text{e}}^{s\Omega }⊗{\text{e}}^{(t-s)\Omega *}⊗{\text{e}}^{(t-s)\Omega *}\text{ds}\;\text{d}\tau \right).$$

If we define the variance-covariance matrix, **Σ**(*t*), of the overall number of cases at time *t* when cases of all types are imported, then we have that:

(13) $$\Sigma (t)=\overset{2m}{\underset{i=1}{\sum }}\text{Var}\left({\text{Y}}^{\left(i\right)}(t)\right).$$

Finally, summing all of the elements of ***V***_*i*_(*t*) and **Σ**(*t*), we obtain the variances $\sigma_{Z^{\left(i\right)}}^{2}(t)$ and $\sigma_{Y}^{2}(t)$ of the total number of cases at time *t* for the processes without and with immigration, respectively:

(14) $$\sigma_{Z^{\left(i\right)}}^{2}(t)=\underset{k,l}{\sum }{\left\{{\text{V}}_{i}\right\}}_{\left(k,l\right)}(t),$$

(15) $$\sigma_{Y}^{2}(t)=\underset{k,l}{\sum }\Sigma_{\left(k,l\right)}(t).$$

### 2.2 Time to Establishment of Invading Strain

We now define the time at which an outbreak is fully established in the resident population. Intuitively, this occurs when the total number of cases in the immigration-free process ***Z***^(*i*)^(*t*) is large enough so that the subsequent epidemic dynamics are well approximated by a deterministic model. At this point of the epidemic, the outbreak also has a low probability of becoming extinct and grows approximately exponentially. We aim to choose a threshold number of cases, *Z*^*∗*^, at which we conclude that this is the case, and interpret this threshold as the number of cases required such that the disease can be said to be *established* in the population.

Without loss of generality, we choose *i* = 1 so that we may drop the superscript *i* and simply refer to the process ***Z***(*t*), starting with a single case of type *i* = 1. We require two conditions for the establishment of an invading strain, namely that the local epidemic is approximately growing according to the mean growth curve and that the probability of having no cases is negligible. We define a (non-random) time *T*^*∗*^ > 0 as the minimum time such that both these criteria are satisfied and, given *T*^*∗*^, we define the threshold number of cases $Z^{*}=\sum_{j=1}^{2m}E\left[Z_{j}\left(T^{*}\right)\right]$ to be the mean number of cases at time *T*^*∗*^.

We formalise these conditions as follows. Define *c*(*t*) := *σ*_*Z*_(*t*)*/µ*_*Z*_(*t*) to be the coefficient of variation for the immigration-free process (which is defined as the standard deviation *σ*_*Z*_(*t*) of the process divided by the mean *µ*_*Z*_(*t*)), and let *q*(*t*) = *P* (*Z*_*j*_(*t*) = 0, 1 ≤ *j* ≤ 2*m*) be the probability that there are no infected individuals of any type at time *t*. Given *ε*_1_, *ε*_2_ > 0, there exist *T*_1_ and *T*_2_, such that:

1. |*c*^′^(*t*)| *< ε*_1_ for *t > T*_1_,
2. |*q*^′^(*t*)| *< ε*_2_ for *t > T*_2_,

where *c*^′^(*t*) and *q*^′^(*t*) denote the time-derivatives of *c*(*t*) and *q*(*t*), respectively. The above conditions ensure that both quantities *c*(*t*) and *q*(*t*) are approximately constant over time. We then choose *T*^*∗*^ = *T*^*∗*^(*ε*_1_, *ε*_2_) such that both of these conditions are satisfied, i.e. *T*^*∗*^ = max{*T*_1_, *T*_2_}. Note that both *ε*_1_ and *ε*_2_, and hence *T*^*∗*^, are deterministic choices to be made. Lower valued choices of *ε*_1_ and *ε*_2_ will result in higher choices of *T*^*∗*^ which, in turn, mean that it is less likely that the disease will go extinct for *t > T* ^*∗*^ and that the coefficient of variation changes less over time; under these conditions the strain is more established and the dynamics can be even better approximated by a deterministic model. We also note that *establishment* could be defined in many alternative ways in different settings and to suit different modelling questions. The choice of definition of *Z*^*∗*^ stated in this section could therefore equally be replaced with a different definition accordingly.

In what follows we will assume, for convenience, that *ε* = *ε*_1_ = *ε*_2_, and refer simply to *ε* without any subscript. Note that in a supercritical process, such that the maximum eigenvalue of **Ω** is greater than 0, both of these conditions are satisfied as *t* → ∞ (see [2, Ch. 7] for details). Whilst we have focused on defining the time to establishment for the process ***Z***(*t*) in the absence of immigration, analogous definitions are straightforwardly obtained for the process ***Y*** (*t*) with immigration. In this case, we would replace *q*(*t*) with *r*(*t*) for the second criterion.

Having defined both *T*^*∗*^ and *Z*^*∗*^, we are interested in finding the distribution of the random variable *T*, which is the First-Passage Time (FPT) of the branching process ***Z***(*t*) to *Z*^*∗*^, defined as:

(16) $$T=\inf\left\{t>0|\overset{2m}{\underset{j=1}{\sum }}Z_{j}(t)=Z^{*}\right\}.$$

### 2.3 One-dimensional Diffusion Model Approximation

In order to study first-passage time distribution for our branching process, we rely on a diffusion approximation, studied by Kurtz [21, 22], but first introduced by Feller [12, 13]. For single-type processes, the authors have previously shown that the Feller diffusion is a good approximation for the first-passage time of an epidemic [6]. For the multi-type process, the Feller diffusion is an example of the multi-dimensional Fokker-Planck equations, and has been studied in, for example, [5, 16, 19, 26].

Recall that **Ω** is the mean offspring matrix for the branching process, whose (*i, j*)^th^ component is the average number of offspring of type *j* produced by an individual of type *i*. Furthermore, let ${\left({\text{G}}_{jk}\right)}_{i}$ be the variance-covariance matrix, i.e. ${\left({\text{G}}_{jk}\right)}_{i}=\text{Var}\left[{\text{ξ}}_{i}\right],$ for the offspring distribution of a type-*i* individual, given by:

(17) $${\left({\text{G}}_{jk}\right)}_{i}=\left(\frac{\partial^{2}P_{i}}{\partial s_{j}\partial s_{k}}\right)-\text{diag}\left(\frac{\partial P_{i}}{\partial s_{j}}\right)+{\text{I}}_{2m},$$

where ***I***_2*m*_ is the 2*m* × 2*m* identity matrix.

For the multi-type process ***Z***(*t*) in the absence of immigration (corresponding to *η* = 0), the Feller diffusion approximation, which we denote by the vector ${\text{X}}_{t}={\left(X_{t}^{\left(i\right)}\right)}_{i=1}^{2m}\approx \text{Z}(t),$, satisfies the vector stochastic differential equation (see [25] for a detailed derivation):

(18) $$\text{d}{\text{X}}_{t}=\Omega {\text{X}}_{t}\text{d}t+\sqrt{\text{C}}\text{d}{\text{W}}_{t},$$

where $C=\sum_{i=1}^{2m}{\left({\text{G}}_{jk}\right)}_{i}X_{t}^{\left(i\right)}$, where $W_{t}=\left(W_{t}^{\left(i\right)}\right)$ is a vector of standard Brownian motions.

To the knowledge of the authors, no analytic solution to (18) is known. However, we may approximate the solution of (18) by projecting the vector ***X***_*t*_ onto the dominant eigenvector of the matrix **Ω**. This approximation will, in turn, offer an approximation of the first passage time of the branching process to the level *Z*^*∗*^ defined previously.

Let ***v***_1_ be the dominant eigenvector of **Ω** associated with the maximum eigenvalue *r*. Following [3], we let $V_{t}≔{\text{v}}_{1}^{T}{\text{X}}_{t}$ so that, by the multivariate version of Itô’s Lemma [20]:

(19) $$\begin{array}{c} \text{d}V_{t}={\text{υ}}_{1}^{T}\text{d}{\text{X}}_{t}={\text{υ}}_{1}^{T}\Omega {\text{X}}_{t}+{\text{υ}}_{1}^{T}\sqrt{\text{C}}\text{d}W_{t}\\ \;=rV_{t}+\sqrt{{\text{υ}}_{1}^{T}\text{C}{\text{υ}}_{1}}\text{d}W_{t}. \end{array}$$

Here, *W*_*t*_ is a standard, one-dimensional Brownian motion. $\xi =\sum {\text{υ}}_{1}^{i}$ be the sum of the components of the dominant eigenvector ***v***_1_. We wish to approximate the distribution of the first-passage time to $Z^{*}=\sum_{j}Z_{j}(t)\approx \sum_{j}{\left({\text{X}}_{t}\right)}_{j}$. Now, since $V_{t}={\text{υ}}_{1}^{T}{\text{X}}_{t}$ we have that $V_{t}=\sum_{j}{\left({\text{X}}_{t}\right)}_{j}/\xi$ and so we may approximate the distribution of the first-passage time to *Z*^*∗*^ with the distribution of the first-passage time of the one-dimensional process *V*_*t*_ to the level *V* ^*∗*^ ≔ *Z*^*∗*^*/ξ*.

We are, as yet, unable to obtain an analytical approximation following a similar argument for the branching process in the case with immigration. To the knowledge of the authors, such an analytical approximation remains an open problem, and is the subject of ongoing research.

### 2.4 First Passage Time Distribution for the Diffusion Approximation

Let *f* (*t, v*) be the probability density function for the number of cases according to the one-dimensional process *V*_*t*_ at time *t*. Also let $\rho ={\text{υ}}_{1}^{T}\text{C}{\text{υ}}_{1}$ and, as before, let *r* be the maximum eigenvalue of the matrix **Ω**. Then the probability density function *f* (*t, v*) has the solution (first derived in [13]):

(20) $$f(t,\upsilon )=\frac{r{\text{e}}^{rt}}{\frac{\rho }{2}\left({\text{e}}^{rt}-1\right)}\sqrt{\frac{{\text{e}}^{rt}}{v}}I_{1}\left(\frac{2r\sqrt{\upsilon {\text{e}}^{rt}}}{\frac{\rho }{2}\left({\text{e}}^{rt}-1\right)}\right)\exp\left(-\frac{r\left({\text{e}}^{rt}+\upsilon \right)}{\rho \left({\text{e}}^{rt}-1\right)}\right),$$

which is the PDF of a non-central *χ*^2^ distribution with zero degrees of freedom, studied by Siegel [29], and where *I*_1_(·) is the modified Bessel function of the first kind. Since the non-central *χ*^2^ distribution with zero degrees of freedom has mass exp $\left\{-\frac{\lambda }{2}\right\}$ at *v* = 0, where $\lambda =4{\text{e}}^{rt}/\left(\rho \left({\text{e}}^{rt}-1\right)\right),$ we must condition on the process not reaching zero in order to obtain a true density function. Hence, we obtain the conditioned density function:

(21) $$\hat{f}(t,\upsilon )=\frac{f(t,\upsilon )}{\left(1-{\text{e}}^{-\frac{\lambda }{2}}\right)},\;\upsilon >0.$$

Finally, the integrated density $\hat{F}(t,\upsilon )=\int_{0}^{\upsilon }\hat{f}(t,x)\text{d}x=\mathrm{Pr}\left(V_{t}\leq \upsilon \right)$ represents the probability that the number of cases has not yet reached the level *v* (conditional on the process not reaching zero cases).

Let *U*_*v*_(*t*) be the cumulative density function (CDF) for the first passage time of the process *V*_*t*_ to the level *v*. The CDF *U*_*v*_(*t*) is obtained from $\hat{F}(t,\upsilon )$ via CDF inversion. Hence, the CDF for the first passage time for the process *V*_*t*_ to reach the level *V*^*∗*^ is given by the formula:

(22) $$U_{V^{*}}(t)=1-\hat{F}\left(t,V^{*}\right)=\mathrm{Pr}\left(T^{*}\leq t\right).$$

The FPT distribution for the process *V*_*t*_ to reach the level *V* ^*∗*^ represents a one-dimensional approximation to the FPT distribution for the total number of cases of all types in the multi-variate process ***Z***(*t*) to reach *Z*^*∗*^. The FPT distribution gives us the times at which the outbreak is established in the population and, at the same time, the time at which it is appropriate to switch to an approximating deterministic model of the population dynamics.

### 2.5 COVID-19 VoC model

As a specific example of our model in practice, we consider a model of the emergence of a COVID-19 variant of concern (VoC) following the successful control of a wild-type strain by a vaccination campaign, along the broad lines of [10]. In particular, we suppose that there is a set of possible components of individuals’ disease histories, *𝒦* = {WT, Vac, VoC}, corresponding to recovery from wild-type infection, vaccination with an early vaccine, and recovery from infection with a VoC, respectively. We do not consider the difference in immunity conferred by vaccines of different types or by different numbers of doses, effectively assuming that all vaccinated individuals have received the same doses of the same type of vaccine. Furthermore, we do not consider any loss of vaccine- or infection-acquired immunity over time. Prior to the emergence of the VoC, the disease dynamics are well approximated by the deterministic set of ordinary differential equations,

(23) $$\begin{array}{lll} \frac{\text{d}}{\text{d}t}S_{\emptyset } & = & -\left(V(t)+\beta \left(I_{\emptyset }^{\text{WT}}+I_{\text{\{Vac\}}}^{\text{WT}}\right)\right)S_{\emptyset },\\ \frac{\text{d}}{\text{d}t}E_{\emptyset }^{\text{WT}} & = & +\beta \left(I_{\emptyset }^{\text{WT}}+I_{\text{\{Vac\}}}^{\text{WT}}\right)S_{\emptyset }-\delta E_{\emptyset }^{\text{WT}},\\ \frac{\text{d}}{\text{d}t}I_{\emptyset }^{WT} & = & \delta E_{\emptyset }^{WT}-\gamma I_{\emptyset }^{WT},\\ \frac{\text{d}}{\text{d}t}S_{\left\{\text{WT}\right\}} & = & \gamma I_{\emptyset }^{\text{WT}}-V(t)S_{\left\{\text{WT}\right\}},\\ \frac{\text{d}}{\text{d}t}S_{\text{\{Vac\}}} & = & V(t)S_{\emptyset }-\sigma_{\text{\{Vac\}}}^{\text{WT}}\beta \left(I_{\emptyset }^{\text{WT}}+I_{\text{\{Vac\}}}^{\text{WT}}\right)S_{\text{\{Vac\}}},\\ \frac{\text{d}}{\text{d}t}E_{\text{\{Vac\}}}^{\text{WT}} & = & +\sigma_{\text{\{Vac\}}}^{\text{WT}}\beta \left(I_{\emptyset }^{\text{WT}}+I_{\text{\{Vac\}}}^{\text{WT}}\right)S_{\text{\{Vac\}}}-\delta E_{\text{\{Vac\}}}^{WT},\\ \frac{\text{d}}{\text{d}t}I_{\text{\{Vac\}}}^{\text{WT}} & = & \delta E_{\text{\{Vac\}}}^{\text{WT}}-\gamma I_{\text{\{Vac\}}}^{\text{WT}},\\ \frac{\text{d}}{\text{d}t}S_{\left\{\text{Vac},\text{WT}\right\}} & = & \gamma I_{\text{\{Vac\}}}^{\text{WT}}+V(t)S_{\left\{WT\right\}}. \end{array}$$

Here, as in Section 2.1 above, *δ* and *γ* are the rates of transitioning from state *E* to state *I* and the recovery rate, respectively, *β* is the baseline rate for transmission of wild-type to an individual with no infection or vaccination history, and $\sigma_{ℐ}^{i}$ represents the relative susceptibility of (i.e. the factor by which *β* should be multiplied for) infection with strain *i* of an individual with disease history ℐ. $S_{ℐ}$ represents the proportion of the total population not currently infected with anything and with disease history ℐ. $A_{ℐ}^{i}$ for *A ∈* {*E, I*} represents the proportion of the total population in disease state *A* (either exposed or infectious), infected with strain *i* and with disease history ℐ. *V* (*t*) denotes the time-varying rate at which individuals who are not in the latent or infectious stage at time *t* are vaccinated. Over time, we assume that the vaccination programme comes to an end, so that lim_*t*→∞_ *V* (*t*) = 0 and it is possible for some individuals to still be in state *S*_*∅*_.

We note that these equations are of a very similar form to the multi-strain equations of [1], except with the restricted set $\tilde{𝒦}=\{\text{WT,Vac}\}\subset 𝒦$ and vaccination behaving differently from infection with the wild-type. Ultimately, the time evolution of System (23) will only leave the *S* variables with the exposed *E* and infectious *I* variables decaying. We will write the final state of the system as

(24) $$\underset{t\to \infty }{\lim}\left(S_{\emptyset }(t),S_{\left\{\text{WT}\right\}}(t),S_{\left\{\text{Vac}\right\}}(t),S_{\left\{\text{Vac,WT}\right\}}(t)\right)=\left(S_{ℐ}^{\infty }\right),\;ℐ\in 2^{\tilde{𝒦}},$$

where $2^{\tilde{𝒦}}$ is the power set – i.e. set of all subsets – of $\tilde{𝒦}$, the elements of which are all possible disease histories prior to the emergence of the VoC.

Against this background, we can then define a multi-type real-time branching process with state vector

(25) $$\text{Z}(t)=\left(Z_{\emptyset }^{E}(t),Z_{\left\{\text{WT}\right\}}^{E}(t),Z_{\left\{\text{Vac}\right\}}^{E}(t),Z_{\left\{\text{Vac},\text{WT}\right\}}^{E}(t),Z_{\emptyset }^{I}(t),Z_{\left\{\text{WT}\right\}}^{I}(t),Z_{\left\{\text{Vac}\right\}}^{I}(t),Z_{\left\{\text{Vac},\text{WT}\right\}}^{I}(t)\right),$$

whose element $Z_{ℐ}^{A}(t)$ for *A ∈* {*E, I*} and $ℐ\in 2^{\tilde{𝒦}}$ corresponds to the random integer-valued number of VoC infections in disease state *A* (either exposed or infectious) and with disease history ℐ (entirely naïve, previously vaccinated, previously infected with wild-type, or both).

This model is a special case of the general model introduced in Section 2.1 above, with an incubation rate *δ* at which each exposed individual becomes infectious, a recovery rate *γ* at which each infectious individual recovers, and infection rates

(26) $$\begin{array}{l} \beta_{1}=\beta \sigma_{\emptyset }^{\text{VoC}}S_{\emptyset }^{\infty }\\ \beta_{2}=\beta \sigma_{\text{\{WT\}}}^{\text{VoC}}S_{\text{\{WT\}}}^{\infty }\\ \beta_{3}=\beta \sigma_{\text{\{Vac\}}}^{\text{VoC}}S_{\text{\{Vac\}}}^{\infty }\\ \beta_{4}=\beta \sigma_{\text{\{Vac,WT\}}}^{\text{VoC}}S_{\text{\{Vac,WT\}}}^{\infty }. \end{array}$$

The multi-type process thus defined captures the early (i.e. linear) stochastic behaviour of a VoC wave happening after a wild-type wave and vaccination. The longer term behaviour of this system, capturing the full non-linearity due to the depletion of susceptibles, will be given by a set of equations similar to those in System (23) and a special case of the model in [10]. Explicitly, for all $ℐ\in 2^{\tilde{𝒦}}$,

(27) $$\begin{array}{lll} \frac{\text{d}}{\text{d}t}S_{ℐ} & = & -\sigma_{ℐ}^{\text{VoC}}\beta S_{ℐ}\underset{J\in 2^{\tilde{𝒦}}}{\sum }I_{J}^{\text{VoC}},\\ \frac{\text{d}}{\text{d}t}E_{ℐ}^{\text{VoC}} & = & \sigma_{ℐ}^{\text{VoC}}\beta S_{ℐ}\underset{J\in 2^{\tilde{𝒦}}}{\sum }I_{J}^{\text{VoC}}-\delta E_{ℐ}^{\text{VoC}},\\ \frac{\text{d}}{\text{d}t}I_{ℐ}^{\text{VoC}} & = & \delta E_{ℐ}^{\text{VoC}}-\gamma I_{ℐ}^{\text{VoC}},\\ \frac{\text{d}}{\text{d}t}S_{ℐ\cup \{\text{VoC}\}} & = & \gamma I_{ℐ}^{\text{VoC}}. \end{array}$$

## 3 Results

### 3.1 Epidemic Model with Heterogeneous Immunity

We consider a concrete implementation of the model described in the previous section, with a full list of parameters given in Table 1, many of which have been taken from [10]. We use the next-generation matrix to calculate the effective reproduction number given the heterogeneous immunity profile in the population [8]. We consider the two cases where importation is not allowed to occur but that where epidemic is seeded by a single exposed individual at time *t* = 0, and the case where importation occurs at an average rate of 1 new exposed case every 10 days, so that *η* = 0.1 (technically, in our model, there is no immigration of external individuals into the population *per se*, but rather members of the population getting infected from an external source at the given rate). In the former case, we assume that the initial exposed case belongs to *type-at-birth* 1, meaning that they have neither infection- nor vaccine-acquired immunity. In the latter case, imported exposed cases can belong to any of the 4 *types-at-birth*, and so for each type, we scale the importation rate by the proportion of individuals of that type in the resident population, giving $\eta_{1}=S_{\emptyset }^{\infty }\eta ,\eta_{2}=S_{\text{WT}}^{\infty }\eta ,\eta_{3}=S_{\text{Vac}}^{\infty }\eta \;\text{and}\;\eta_{4}=S_{\text{\{Vac,WT\}}}^{\infty }\eta$.

**Figure 1:**
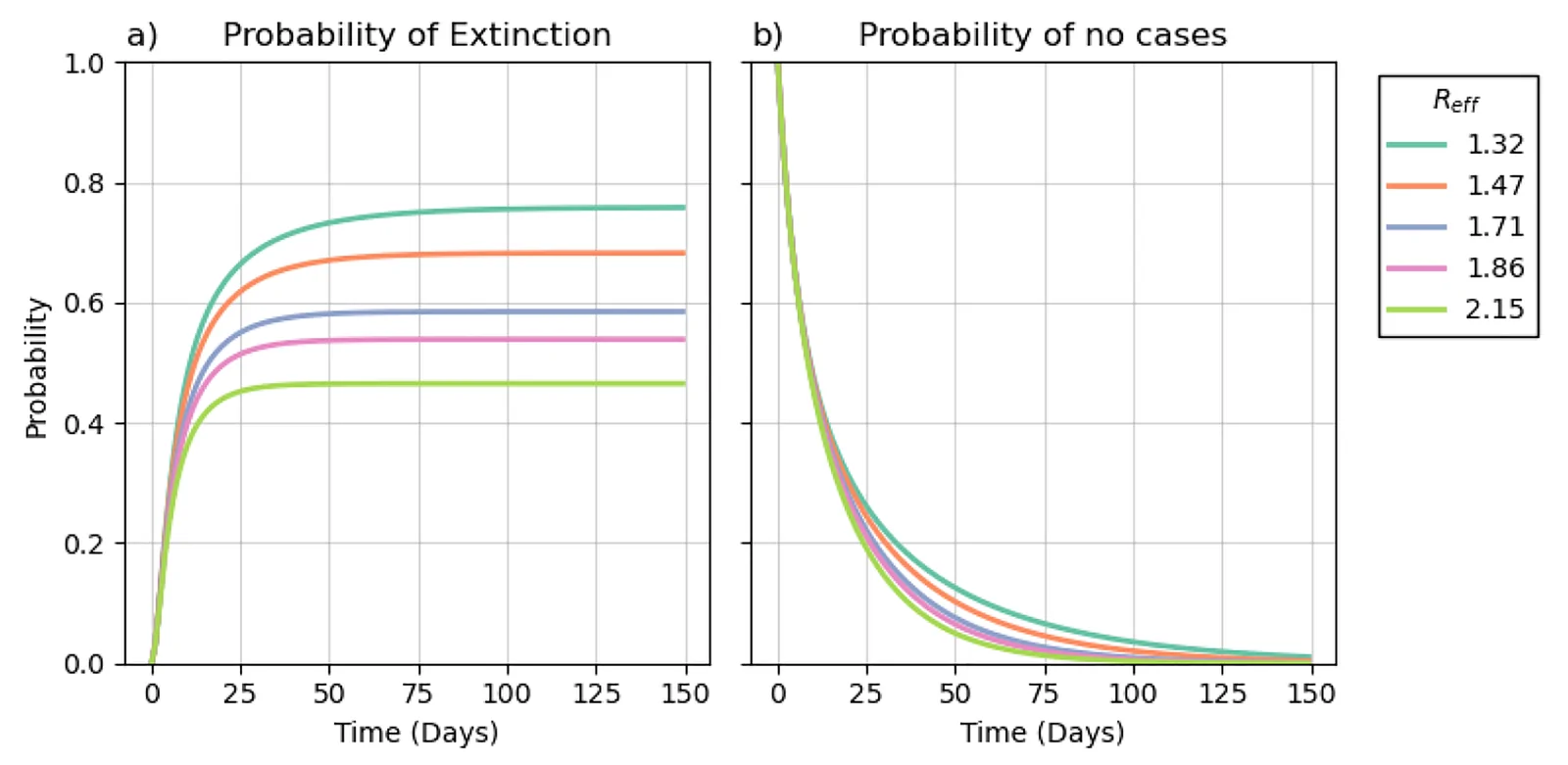
a) Probability that a transmission chain beginning with a single exposed case of type 1 (i.e. neither vaccinated nor infected with a previous strain) goes extinct before time *t*, in the absence of importation of cases. These probabilities tend to a constant value *q*_∞_ in the limit as *t* → ∞. b) Probability that there are no cases of any type at time *t* when there are zero cases at time *t* = 0 and importation occurs at a constant rate *η* = 0.1. These probabilities all tend to zero as *t* → ∞.

We show the probability that, at time *t*, there are no cases of any type in Figure 1. We consider separately the extinction probability for a transmission chain that begins with a single imported case of the VoC and for the total number of cases of an invading VoC starting from no initial case, but with importation occurring at a rate *η* = 0.1. Since *R_eff_* > 1 for all of the scenarios we consider, the extinction probability for any individual transmission chain always tends to a limit that is lower than 1. When importation of cases is allowed to occur, these probabilities will always tend to zero over time, as eventually at least one of the outbreaks started by an imported case will not go extinct.

### 3.2 Time to Establishment of an Outbreak

By exploring different values of *R_eff_* for the VoC we can use the time to establishment of a VoC given by *T*^*∗*^ as a measure of the effectiveness of control policies aimed at reducing transmission in the resident population. Similarly, by exploring different values of *η* we can assess the impact of intervention that reduce importation of the infection into the population, e.g. border controls. We assess the impact of measures taken against a newly emerging strain both in the resident population and at the border on the time to establishment of a VoC as measured by *T*^*∗*^. We begin by considering the mean and coefficient of variation for the multi-type process as described in Section 2.1. We then choose *T*^*∗*^ based on the criteria defined in Section 2.2, which we show in Figure 3. As we expect, as the value of *R_eff_* increases, the time *T*^*∗*^ required for both of our conditions 1 and 2 to be satisfied decreases, reflecting the fact that an outbreak reaches a large number of cases more quickly.

For *R_eff_* = 1.32, we obtain a choice of *T*^*∗*^ = 173 days (taking *ε* = 10^−3^ in Conditions 1 and 2 in Section 2.2) for a transmission chain resulting from a single initial case of type 1, compared with *T*^*∗*^ = 95 days for an outbreak in which importation is allowed to occur at a rate *η* = 0.1, but that starts with no cases at time *t* = 0. When *R_eff_* = 2.15, we obtain a choice of *T*^*∗*^ = 55 days in the absence of importation, and *T*^*∗*^ = 69 days when importation is included. For this higher value of *R_eff_*, the probability of having zero cases tends to zero more slowly in the case where importation is included, because the process in this case starts with zero cases. This results in a larger value of *T*^*∗*^ for the chosen value of *ε* = 10^−3^, compared with the case without immigration but starting with a single exposed individual. The large time taken for a strain to become established when *R_eff_* is closer to 1 reflects the fact the variation around the mean growth curve remains large, given higher proportion of realisations of the process having low growth for extended periods of time. The impact of importation on *T*^*∗*^ is therefore much more significant when *R_eff_* is close to one.

In general, increasing border restrictions (and, thus, lowering the importation rate *η*, see bottom right of Figure 3) has the effect of increasing the time *T*^*∗*^ until the novel strain becomes established in the population. This reflects the intuition that border controls can be seen as a mechanism for “buying time” to prepare other necessary interventions for mitigating the impact of a novel VoC on the resident population. Furthermore, we note that even small increments in the importation rate have the same detrimental effect as a substantial increase in *R_eff_*: e.g. an absolute increment in *η* of 0.025 (from *η* = 0.075 to *η* = 0.1) has an equivalent effect of increasing *R_eff_* from roughly 1.3 to 2 or more. Even small changes at very low values (e.g. from 0.05 to 0.075) correspond to large differences in the time it takes for a novel strain to become established. Furthermore, for relatively high importation rates the impact of *R_eff_* on the time to establishment becomes almost negligible. This is in line with previous insight that significant delays in the time it takes for a pathogen to invade require extremely low importation rates [27, 18].

### 3.3 First Passage Time Distribution

**Figure 2:**
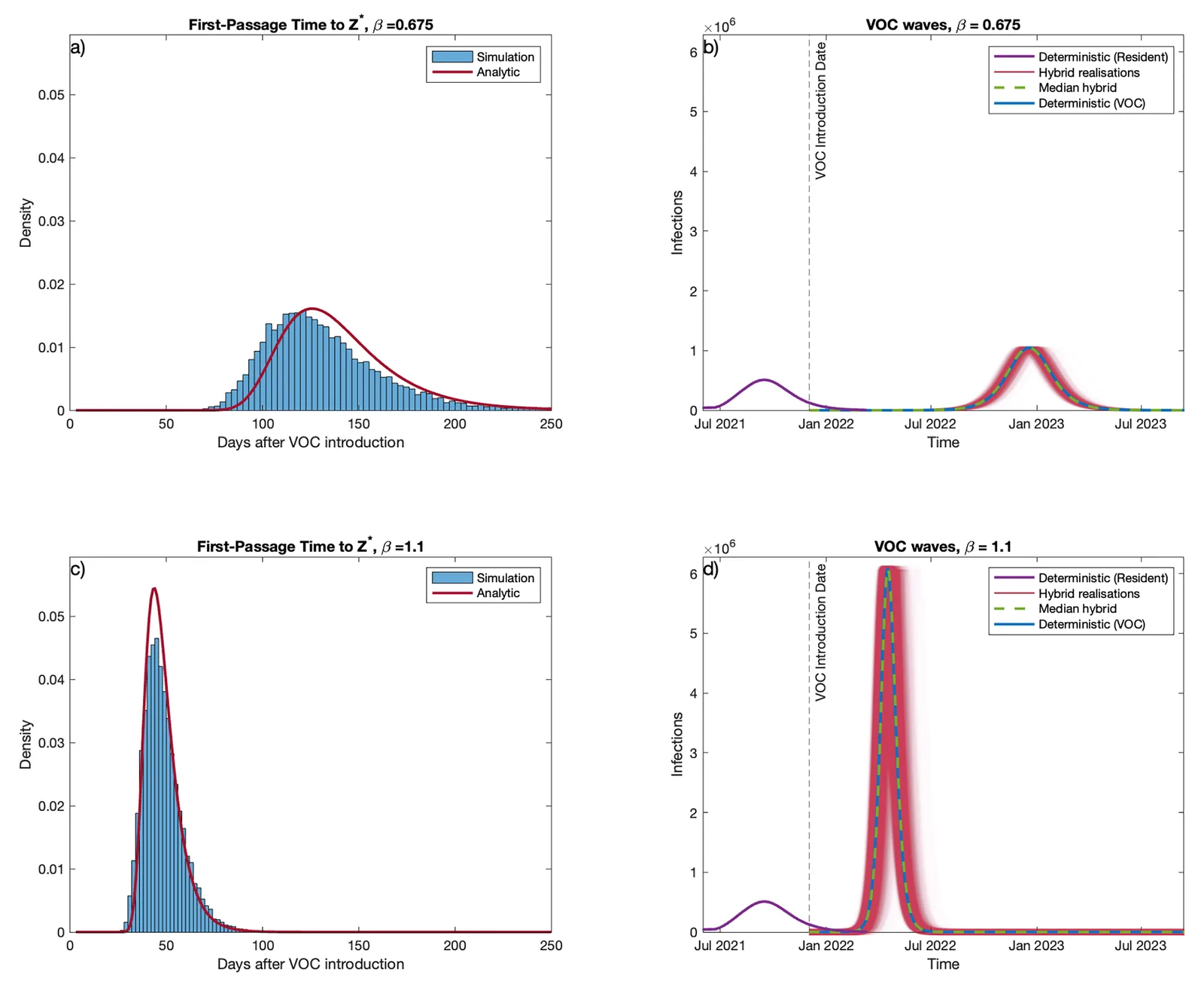
Estimated first-passage time distributions for the full multi-type branching process model based on 10^4^ trajectories simulated using the Gillespie algorithm, compared with the exact FPT distribution of the one-dimensionalised diffusion approximation (a) for *R_eff_* = 1.32 and (c) for *R_eff_* = 2.15. *Right column:* Modelled waves of a novel VoC in the UK, comparing the deterministic solution of the compartmental model with hybrid realisations of the deterministic model, randomly shifted in time according to the FPT distribution (b) for *R_eff_* = 1.32 and (d) for *R_eff_* = 2.15.

**Figure 3:**
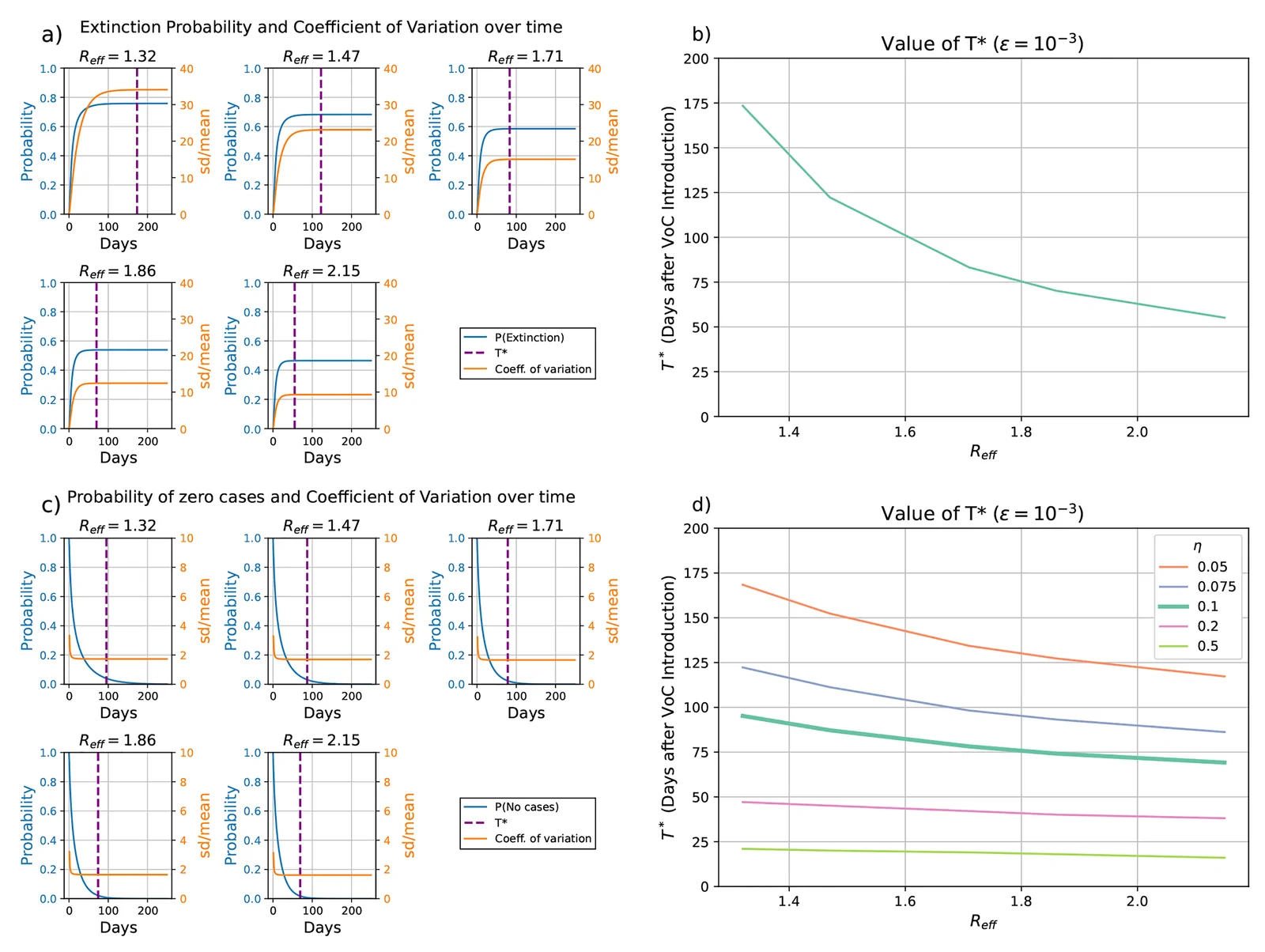
a) Probability of zero cases and coefficient of variation over time for an outbreak without importation of cases, for different values of *R_eff_*, as well as the value of *T*^*∗*^ obtained from Conditions 1 and 2 in Section 2.2 for *ε* = 10^−3^ shown in purple. b) Summary of the value of *T*^*∗*^ as a function of *R_eff_* for an outbreak without importation of cases, which clearly shows a monotonic decrease. c) Probability of zero cases and coefficient of variation over time for an outbreak that begins with no cases, but allows importation of cases at a baseline rate *η* = 0.1. d) Summary of the value of *T*^*∗*^ as a function of *R_eff_* for an outbreak that begins with no cases and with importation of cases at different rates, *η*.

To test the quality of our analytical approximation to the FPT distribution, we first simulate the true FPT distribution of the full multi-type branching process with offspring distribution given by Equation (2) using the Gillespie algorithm [15]. Given we could only develop the analytical approximation in the absence of importation, we restrict results to this case. We simulate 10,000 trajectories from the branching process and record the first time at which the total number of cases passes the threshold *Z*^*∗*^. The resulting distribution is shown in the left panels in Figure 2.

We then compare the simulation output from the Gillespie algorithm with the probability density function for the approximated FPT distribution obtained using the one-dimensionalisation of the multi-type Feller diffusion given in Equation (22). These comparisons are shown in Figure 2 for *R_eff_* = 1.32 and for *R_eff_* = 2.15. In both cases, we find that the one-dimensional approximation based on the Feller diffusion provides a very good approximation of the true underlying distribution, estimated via simulations. As can be expected, the one-dimensional Feller approximation is closer to the true distribution for higher values of *R_eff_*, reflecting the fact that convergence to the one-dimensional process is improved for higher values of the dominant eigenvalue, *r*.

### 3.4 COVID-19 VoC Model

Finally, we apply our results for the first-passage time distribution to the emergence of a novel VoC in the UK, beginning with a single case on the 2^nd^ of December 2021, as done in [10]. This occurs against the background of an outbreak of a resident strain that, by the time of the invasion of the novel strain, has infected 46.1% of the population and a partially complete vaccination programme, such that 74.6% of individuals have received a full course of vaccination (i.e., two doses). The proportion of people in the population who, at the end of the resident strain outbreak, have both been previously exposed to the resident strain and have been vaccinated is 30.1%. These proportions are outputs obtained by solving System (23) using parameter values taken from [10] and taking *t* → ∞ to obtain the final states given in Equation (24). The rate *V* (*t*) at which individuals in the population become vaccinated is taken from the rollout speed scenario given to the UK Cabinet Office given in [28].

Following the introduction of the VoC, we model the growth, peak and decay of the resulting epidemic according to System (27). In addition to this, we model the early phase of the outbreak using a multi-type branching process described by Equation (25) until the epidemic reaches a level $Z^{*}=\sum_{j=1}^{8}E\left[Z_{j}\left(T^{*}\right)\right],$, where *T*^*∗*^ is derived by satisfying Conditions 1 and 2 in Section 2.2 for *ε* = 10^−3^. Once this number of cases has been achieved, we generate 1,000 realisations of the epidemic by generating a random start time from the first-passage time distribution to *Z*^*∗*^. At each randomly selected start time, we generate 1,000 realisations of the epidemic by first solving System (27) starting with *Z*^*∗*^ cases, which are distributed among the types proportionally to the components of the principal eigenvector of **Ω, *v***_1_, and then translating 1,000 copies of this solution such that each copy starts at a random time generated from the first-passage time distribution to *Z*^*∗*^. In Figure 2 we show the comparison between the hybrid realisations and the continued deterministic trajectory of the epidemic curve for the VoC. We note that the hybrid realisation that starts at the median first-passage time to *Z*^*∗*^ shows good agreement with the continued deterministic trajectory for both low and high values of *R_eff_*, demonstrating that our approximation accurately captures both the average peak time, as well as the distribution of peak times.

## 4 Discussion and Conclusions

We have used a multi-type branching process model of the early phase of linear growth of an epidemic to calculate the mean and variance in the number of cases at a given time. We have defined the time at which an outbreak has become established in the population using a set of criteria based on the probability over time that the disease goes extinct and on the coefficient of variation for the prevalence of the disease. We have chosen this time such that future extinction of the strain is unlikely and such that the standard deviation is growing exponentially and proportionally to the mean. We have then quantified the uncertainty in this time, which we have interpreted as the time taken for a novel strain to become successfully established in the population, by using simulations to explore the first-passage time distribution. For a single initial case whose transmission chain does not go extinct, and in the absence of further importation of cases, we have used a one-dimensional Feller diffusion approximation to obtain an analytic expression that approximates such a first-passage time distribution for the time taken for the VoC to be successfully established in the population. As an example of this model in practice, we have considered an outbreak of a novel variant of concern in a population with prior exposure to previous variants of SARS-CoV-2 that has undergone a partial vaccination programme, following the analysis undertaken in [10].

Our results demonstrate the practical utility of using one-dimensional Feller diffusion approximation to the multi-type branching process as an alternative to simulating the FPT distribution. In this work we have only been able to derive such an approximation of the FPT distribution of the branching process in the case without importation of cases. In these cases, a key strength of our approach is that it drastically reduces the computation time required compared with simulations performed using the Gillespie algorithm, given that drawing random numbers from the FPT is almost instantaneous. The diffusion approximation also offers additional insight into the true underlying distribution, suggesting that the total number of cases at a given time *t* in a multi-type branching process is well approximated by a non-central *χ*^2^ distribution with zero degrees of freedom. Our approximation therefore lends itself well to modelling scenarios that need to be run multiple times for many different parameter choices, when running large numbers of simulations is computationally unfeasible.

When modelling the importation of a novel VoC into the UK, we have assumed that infection- and vaccination-acquired immunity only impact the susceptibility of individuals and not the transmissibility of infectious individuals, so that our model outputs can be more easily compared with modelling work that was undertaken in [10]. However, the general method we have presented in this work would easily allow for this assumption to be relaxed and for a different transmission rate to be assigned to each infectious type. We further expect that the quantitative insights gained by including some moderate heterogeneity in transmission across types would not change drastically from those we have presented here, and that the quality of the one-dimensional Feller diffusion approximation should remain high.

One limitation of our work is that we do not consider the impact that waning immunity against the novel VoC (due to infection or vaccination) could have on the longer-term dynamics of the epidemic. This would not be expected to affect the stochastic part of our model, which is concerned with the early-growth phase of the epidemic. We acknowledge a term governing loss of immunity could be straightforwardly incorporated into the deterministic equations. However, we did not include this aspect to keep focus on the main objects of study, namely the delays and probability of extinction associated with the early stochastic phase of the epidemic.

An additional limitation of our approach is that we are currently unable to obtain an approximate FPT distribution when importation occurs at a rate *η >* 0, which introduces a positive constant drift term to the one-dimensional Feller diffusion. However, solving the SDE in Equation (18) with immigration included is the subject of ongoing work.

The insights for policymakers gained from our modelling complement the work in [18] and [27], which emphasise the difficulty in relying strategies aimed at preventing the incursion of diseases into a population. In our approach, we interpret the efficacy of border controls and internal non-pharmaceutical interventions in terms of delaying the ingress and establishment of novel pathogenic strains in a population. Our model output therefore provides policymakers with a time window in which a novel strain is likely to become established in the population, which allows for any appropriate measures to be planned in response.

## Figures and Tables

**Table 1:** Chosen epidemiological parameters, taken from [10]. *R_eff_* is calculated using the Next-Generation matrix [8].

Parameter	Explanation	Values
*γ*	Recovery rate.	4 days^−1^
*δ*	Incubation rate.	3 days^−1^
*ε(=ε*_1_ = *ε*_2_)	Threshold for choosing T*, and hence Z*.	10^−3^
*β*	Baseline transmission rate.	0.675, 0.75, 0.875, 0.95, 1.1
*R*_0_	VoC Reproduction Number excluding immunity.	2.7, 3.0, 3.3, 3.8, 4.4
*R_eff_*	VoC Effective Reproduction Number.	1.32, 1.47, 1.71, 1.86, 2.15
*t*_VoC_	VoC Introduction date.	2^nd^ December 2021
*Z*_0_	Initial number of VoC infections (in the models with or without importation of cases).	1 (without) or 0 (with)
*η*	VoC Importation rate.	0 or 10 days^−1^
$S_{\emptyset }^{\infty }$	Proportion of individuals neither vaccinated, nor ever infected with a previous strain.	Modelled
$S_{\text{\{Vac\}}}^{\infty }$	Proportion of individuals vaccinated but never infected with a previous strain.	Modelled
$S_{\text{\{Vac\}}}^{\infty }$	Proportion recovered from a previous (wild-type) strain.	Modelled
$S_{\text{\{Vac,WT\}}}^{\infty }$	Proportion both vaccinated and recovered from a previous strain.	Modelled
$\sigma_{\emptyset }^{\text{VoC}}$	Relative susceptibility to VoC of individuals with no history of vaccination or infection.	1
$\sigma_{\text{\{WT\}}}^{\text{VoC}}$	Relative susceptibility to VoC of individuals recovered from a previous strain.	0.6
$\sigma_{\text{\{Vac\}}}^{\text{VoC}}$	Relative susceptibility to VoC of vaccinated in-dividuals.	0.4
$\sigma_{\text{\{Vac,WT\}}}^{\text{VoC}}$	Relative susceptibility to VoC of both recovered and vaccinated individuals.	$\min\left(\sigma_{\left\{\text{Vac}\right\}}^{\text{VoC}},\sigma_{\left\{\text{WT}\right\}}^{\text{VoC}}\right)=0.4$

## Data Availability

The code needed to reproduce the results shown here is available at https://github.com/JCurran-Sebastian/MultiType_Branching_COVID19 and at 10.5281/zenodo.14014377.
